# A Novel Humanized GLP-1 Receptor Model Enables Both Affinity Purification and Cre-LoxP Deletion of the Receptor

**DOI:** 10.1371/journal.pone.0093746

**Published:** 2014-04-02

**Authors:** Lucy S. Jun, Aaron D. Showalter, Nosher Ali, Feihan Dai, Wenzhen Ma, Tamer Coskun, James V. Ficorilli, Michael B. Wheeler, M. Dodson Michael, Kyle W. Sloop

**Affiliations:** 1 Endocrine Discovery, Lilly Research Laboratories, Eli Lilly and Co., Indianapolis, Indiana, United States of America; 2 Department of Physiology, University of Toronto, Toronto, Ontario, Canada; CRCHUM-Montreal Diabetes Research Center, Canada

## Abstract

Class B G protein-coupled receptors (GPCRs) are important regulators of endocrine physiology, and peptide-based therapeutics targeting some of these receptors have proven effective at treating disorders such as hypercalcemia, osteoporosis, and type 2 diabetes mellitus (T2DM). As next generation efforts attempt to develop novel non-peptide, orally available molecules for these GPCRs, new animal models expressing human receptor orthologs may be required because small molecule ligands make fewer receptor contacts, and thus, the impact of amino acid differences across species may be substantially greater. The objective of this report was to generate and characterize a new mouse model of the human glucagon-like peptide-1 receptor (hGLP-1R), a class B GPCR for which established peptide therapeutics exist for the treatment of T2DM. *hGLP-1R* knock-in mice express the receptor from the murine *Glp-1r* locus. Glucose tolerance tests and gastric emptying studies show *hGLP-1R* mice and their wild-type littermates display similar physiological responses for glucose metabolism, insulin secretion, and gastric transit, and treatment with the GLP-1R agonist, exendin-4, elicits similar responses in both groups. Further, ex vivo assays show insulin secretion from humanized islets is glucose-dependent and enhanced by GLP-1R agonists. To enable additional utility, the targeting construct of the knock-in line was engineered to contain both flanking LoxP sites and a C-terminal FLAG epitope. Anti-FLAG affinity purification shows strong expression of hGLP-1R in islets, lung, and stomach. We crossed the *hGLP-1R* line with *Rosa26Cre* mice and generated global *Glp-1r^−/−^* animals. Immunohistochemistry of pancreas from humanized and knock-out mice identified a human GLP-1R-specific antibody that detects the GLP-1R in human pancreas as well as in the pancreas of *hGLP-1r* knock-in mice. This new *hGLP-1R* model will allow tissue-specific deletion of the *GLP-1R*, purification of potential GLP-1R partner proteins, and testing of novel therapeutic agents targeting the hGLP-1R.

## Introduction

The gluco-regulatory actions of the glucagon-like peptide-1 (GLP-1) and its related therapeutic mimetics include enhancement of glucose-dependent insulin secretion and suppression of glucagon release from pancreatic islets, as well as a decrease in postprandial glucose excursion by delay of gastric emptying and centrally-mediated effects on satiety and energy metabolism [1], [2]. These physiological effects are mediated by the GLP-1 receptor (GLP-1R) and have been determined as a result of iterative basic, translational, and clinical experimentation. Important early findings in incretin biology include proof-of-concept studies showing GLP-1 infusion improves hyperglycemia in patients with type 2 diabetes [3] and cloning of the GLP-1R cDNA [4]. In addition, discovery of the dipeptidyl peptidase-4 (DPP4)-resistant GLP-1R agonist exendin-4 (EX-4) enabled much of our current understanding of GLP-1R activation on glucose homeostasis via studies in animal models [5], [6]. Further, clinical investigations of GLP-1 mimetics demonstrated pleiotropic anti-diabetic pharmacology in humans [7], [8].

While the insulinotropic actions of GLP-1 are well characterized, functional roles of the GLP-1R in extra-pancreatic tissues are less understood. GLP-1 treatment affects hemodynamics in both rodents and humans [9], [10]. However, the GLP-1R-expressing cell types that mediate these effects are not known, although recent studies showed atrial GLP-1R-mediated regulation of blood pressure in mice [11]. In addition, while lung Glp-1r mRNA expression is high in some species [12] and GLP-1R signaling is implicated in surfactant secretion [13]–[15], the role of GLP-1R in pulmonary function is not well established. Further, GLP-1 effects on adiposity and lipid metabolism are not completely understood. Although it is well known that GLP-1R agonists inhibit food intake and reduce body weight, *Glp-1r* ^−/−^ mice are protected from high fat diet-induced obesity and insulin resistance and have decreased hepatosteatosis [16]–[18]. Finally, the role of GLP-1 in the thyroid gland remains to be determined, although GLP-1R expression analyses indicate this is largely a rodent phenomenon [19], [20].

To further characterize GLP-1R, it is important to understand which organs and cell types express the receptor. Glp-1r mRNA is present in many tissues with pancreas, lung, stomach, and small intestine showing the highest levels [21]–[23]. Unfortunately, determining GLP-1R protein expression patterns has been technically difficult due to lack of quality GLP-1R antisera. This has been highlighted in reports demonstrating several commercially available GLP-1R antibodies show non-specific staining in cells not expressing Glp-1r mRNA [16], [24]. Developing better reagents and quality control strategies is needed to determine GLP-1R-positive cell types. Once the expression pattern of GLP-1R is firmly established, targeting strategies to assess GLP-1R function in individual tissues will evaluate the extra-pancreatic physiology of the GLP-1R.

Here, we describe a novel mouse line generated via homologous recombination that expresses human *GLP-1R* (*hGLP-1R*) from the murine *Glp-1r* locus. This approach enables immunohistochemistry (IHC) analyses using an anti-GLP-1R antibody that specifically recognizes hGLP-1R. A unique aspect of this model is the inclusion of an eight amino acid (aa) FLAG epitope fused in-frame at the C-terminus of *hGLP-1R*. This feature enables anti-FLAG IHC, western blot (WB), and immunoprecipitation (IP)/affinity purification experiments. In addition, the *hGLP-1R* is flanked by LoxP sites that allow conditional deletion of the *hGLP-1R* using P1 bacteriophage Cre recombinase [25]. We demonstrate initial utility of this element by crossing animals with *Rosa26Cre* transgenic mice [26] to generate whole-body *Glp-1r^−/−^* animals. Use of anti-human GLP-1R and anti-FLAG immunodetection applications with tissues from *hGLP-1R* and *Glp-1r^−/−^* mice enables well-controlled studies of GLP-1R expression. Future studies using this model will facilitate *ex vivo* biochemical characterization of GLP-1R regulation via affinity purification procedures and *in vivo* investigation of GLP-1R function by temporal and conditional gene deletion.

## Materials and Methods

### Ethics Statement

Animals were studied and maintained in accordance with the Institutional Animal Care and Use Committee (IACUC) of Eli Lilly and Company, the Guide for the Use and Care of Laboratory Animals by the National Institutes of Health, and the German Animal Welfare Act. All animal studies described herein were approved by the IACUC of Eli Lilly and Company.

### Animal Husbandry

Mice were singly housed in microisolator cages on wood chip bedding with food (2014 Teklad Global Diet, Harlan, Indianapolis) and deionized water available *ad libitum*. Lights were on a 12∶12 hour light: dark cycle (except when noted otherwise), and temperature and relative humidity were maintained between 21 and 23°C and 45 and 65%, respectively. Ill or moribund animals were sacrificed using CO_2_ asphyxiation and post-mortem cardiac blood draw and/or cervical dislocation; the same methods of euthanasia were applied to animals at the end of any in vivo studies.

### Generation of hGLP-1R and Glp-1r ^−/−^ Mice

Both mouse lines were generated at TaconicArtemis GmbH (Cologne, Germany). The targeting vector to create the models is based on a 10.5-kb genomic fragment from the murine *Glp-1r* (*mGlp-1r*) gene encompassing exons 1 to 2 and flanking sequences. This fragment, obtained from the C57BL/6J RP23 BAC library, was modified by replacing the majority of the coding region of *mGlp-1r* with an engineered *hGLP-1R* cDNA. The C57BL/6NTac ES cell line was grown on an antibiotic resistant, mitotically inactivated feeder layer comprised of mouse embryonic fibroblasts. 1×10^7^ cells and 30 μg of linearized DNA targeting vector were electroporated (Bio-Rad Gene Pulser) at 240 V and 500 μF. Double positive selection with G418 (200 μg/mL) and 1.5 μg/mL Puromycin started on day 2. Correctly recombined ES cell clones were identified by Southern blot analysis using external and internal probes and were frozen in liquid nitrogen. Superovulated BALB/c females were mated with BALB/c males, and blastocysts were isolated from the uterus at days post coitus 3.5. A microinjection pipette was used to inject 10–15 targeted C57BL/6NTac ES cells into each blastocyst. Eight injected blastocysts were implanted into pseudopregnant NMRI females.

*hGLP-1R* knock-in and *Glp-1r^−/−^* embryos were cryopreserved and shipped to the U.S. for re-derivation at Taconic, USA (Germantown, NY). Genomic DNA was extracted from tail clips using the NucleoSpin Tissue kit (Macherey-Nagel), and PCR was used to genotype animals with PCR amplicons analyzed using a Caliper LabChip GX device. The genotyping primer sequences are listed in Table S1. The genotyping strategies are listed in Figures S1 and S2.

### In vivo and in vitro Studies

All animals were randomized into their respective treatment groups based on body weight and were males between 8–12 weeks age. We used n = 7–9 per group to allow for strength of statistical outcomes and each in vivo study was replicated three times. Glucose tolerance tests were performed as previously described [27]. Detailed protocols for islet studies as well as the cAMP accumulation assays are previously described [27], [28]. For gastric emptying, animals housed in reverse light cycle were fasted overnight and then allowed to eat a pre-weighed portion of food for one hour after the start of the dark cycle the next morning. After food consumption was measured, EX-4 (10 nmol/kg body weight (BW)) was injected subcutaneously (SC). The mice were euthanized one hour after EX-4 administration. Stomachs were removed and weighed full and empty for measurement of percent gastric emptying. The mixed meal tolerance test (MMTT) was performed as previously described with some minor modifications [29]. Briefly, all mice were fasted for 4 hours in the morning of the test. At 0 minutes, the animals were orally gavaged with 10 m1/kg BW of Ensure Plus (Abbott). Blood glucose and serum insulin were measured at 0, 10, 30, 60, and 120 minutes post-gavage, and averaged for each group of animals. For IHC, tissues and cells were fixed and processed for paraffin embedding using routine procedures as described previously [30]. Paraffin-embedded human pancreas samples were purchased from Folio Biosciences (Columbus, OH).

### IP-WB and mRNA Expression Analyses

Animals were euthanized using CO_2_ and tissues were harvested and frozen in liquid nitrogen. Frozen tissue (5 mg) was lysed in RIPA buffer using the FastPrep Lysing Matrix system (MpBio). The anti-FLAG M2 antibody (F3165, Sigma) was coupled to MyOne Tosylactivated Dynabeads (65501, Invitrogen by Life Technologies) according to manufacturer protocols. Lysates were incubated with the anti-FLAG M2 antibody-coupled beads overnight at 4°C. Beads were pelleted, washed, PNGase F-treated (G5166, Sigma) (if applicable), and proteins eluted in SDS sample buffer/2.5% mercaptoethanol. WB’s were performed using 10% PAGE followed by dry transfer to nitrocellulose membrane and probed with anti-FLAG L5-HRP antibody (NBP1-06712H, Novus; 1∶10,000 in 0.5% non-fat dry milk) and enhanced chemiluminescence (ThermoFisher). GLP-1R mRNA expression analyses was performed by RT- and qPCR as previously described [27]. The primer and probe sequences are listed in Table S1.

### Statistical Analysis

All results are presented as mean±SEM with all data and statistical analyses performed using Prism4 software (GraphPad).

## Results

**Figure 1:**
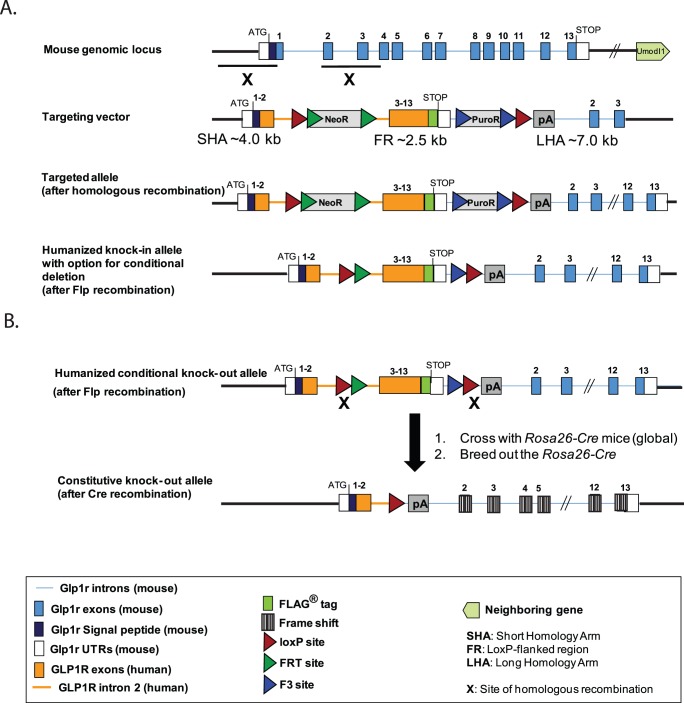
Strategies to generate *hGLP-1R* knock-in and *Glp-1r^−/−^* mice. (A) The targeting vector was injected into ES cells derived from a C57BL/6 line and implanted into BALB/c females, allowing generation of pure C57BL/6 offspring. The targeting construct was designed to insert into the downstream region of exon 1 of the mouse *Glp-1r* genomic locus. Chimeric male offspring were bred to C57BL/6-Tg (CAG-Flpe)2 Arte females that ubiquitously express Flp recombinase to give rise to the final *hGLP-1R* mouse line with option for conditional deletion. The humanized allele contains human GLP-1R cDNA with a preserved human intron 2, a region containing potential regulatory elements conserved across species. (B) *Glp-1r^−/−^* mice were generated by breeding *hGLP-1R* animals to *Rosa26-Cre*-transgenic mice. An additional breeding step was performed to remove the *Cre* gene, resulting in the *Glp-1r^−/−^* line.

**Figure 2:**
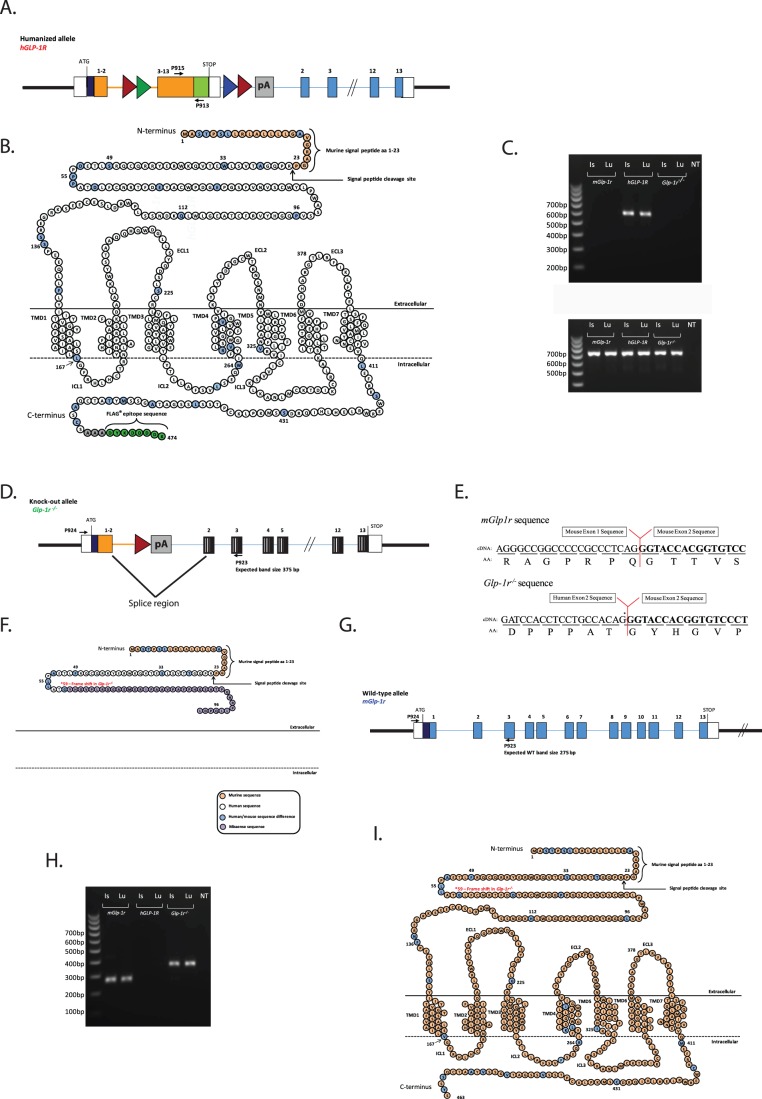
Reverse transcription for PCR validation of *hGLP-1R, mGlp-1r* and *Glp1r^−/−^* lines. (A) A schematic of the gene that is expressed in the *hGLP-1R* mice. (B) The protein that is produced from this gene is a fusion of the mouse signal peptide (beige residues) and the human GLP-1R protein (white residues). The blue residues are those that differ between mouse and human GLP-1R. The signaling peptide is cleaved, leaving behind a human GLP-1R protein containing the C-terminal FLAG epitope (green residues). (C) cDNA was generated from total RNA isolated from islet and lung of *hGLP-1R, mGlp-1r*, and *Glp1r^−/−^* mice for RT-PCR. The 5′ P915 primer annealed in human exon 8, while the 3′ P913 primer annealed to the FLAG region, a unique site within the *hGLP-1R* gene. This PCR product is a 588 bp fragment only observed in the *hGLP-1R* mice. (D) A schematic of the gene that is expressed in the *Glp-1r^−/−^* mice. (E) Once the splice event occurs between human exon 2 and mouse exon 2, a frame-shift mutation nullifies downstream protein expression. (F) The final protein product in *Glp-1r^−/−^* mice is a 98-aa truncation mutant. The first 36 aa’s of the mature protein encode a fraction of the GLP-1R extracellular domain, and the remaining 40 aa’s constitute missense sequence that shows no similarity to known proteins. The RT-PCR and DNA sequence analyses of the *Glp-1r^−/−^* gene product demonstrate the *Glp-1r^−/−^* mouse does not code for a functional GLP-1R. (G) A schematic of the wild-type (*mGlp-1r*) gene. (H) Using the same primer pair, PCR products from *Glp-1r^−/−^* (372 bp) and *mGlp-1r* (275 bp) mice differ in size by 97 bp. (I) The wild-type GLP-1R protein is 463 aa’s including the signaling peptide.

The targeting vector was designed to include several attributes that lend utility and experimental flexibility. The *hGLP-1R* mouse, generated via homologous recombination, expresses a modified *hGLP-1R* cDNA containing a C-terminal FLAG epitope (**Figure 1A**). The *hGLP-1R* is expressed from the murine *Glp-1r* promoter and endogenous upstream regulatory elements. In an effort to maintain normal processing and intracellular trafficking, the *mGlp-1r* 5′ UTR, start codon, and signal peptide sequences remain in place and are immediately followed by exon 2 of the *hGLP-1R*; upon transport to the plasma membrane and subsequent proteolytic cleavage of the murine signal peptide, the mature protein is 100% human GLP-1R with a C-terminal FLAG fusion (**Figure 2B**). The FLAG tag, engineered prior to the stop codon, enables anti-FLAGFLAG antibody methodologies. In order to delete *hGLP-1R*, flanking LoxP sites were inserted upstream of human exons 3–13 and downstream of the mouse 3′ UTR (**Figure 1A**). Additionally, the polyA signal sequence from the human *growth hormone* gene was inserted downstream of the distal LoxP site to prevent transcriptional read-through (**Figure 1A**).

Once the *hGLP-1R* mouse line was created, animals were bred with *C57BL/6-Gt(ROSA)26Sortm16(Cre)* mice for germ-line deletion of *hGLP-1R* (**Figure 1B**). The *Rosa26* locus enables global transgene expression, and *Rosa26 Cre* mice ubiquitously express the Cre enzyme [31]. Deletion of *hGLP-1R* results in loss of function by removing most of the coding sequence (**Figure 1B**) and generating a frame-shift mutation in the downstream mouse *Glp-1r* gene, obviating translation of the remaining *mGlp-1r* (**Figure 1B**). To remove the *Rosa26 Cre* locus, *Rosa26 Cre:Glp-1r^−/−^* offspring were crossed with wild-type C57BL/6 mice to obtain *Glp-1r^+/−^* offspring that were used as breeders to generate all cohorts for these studies. Schematics indicating the GLP-1R aa sequences in each genotype (**Figures 2B, 2F, 2I**) are illustrated similar to the depiction by Doyle and Egan [32].

Total RNA from islets and lung tissue of *mGlp-1r*, *hGLP-1R*, and *Glp-1r^−/−^* mice was isolated for RT-PCR and sequencing analyses (**Figure 2**). Expression of the *hGLP-1R* allele was detected using the primer set P915/P913 that amplifies a region between exon 8 and the FLAG epitope (**Figure 2A**). The resulting product is a single 588 bp band that is only amplified in the *hGLP-1R* line tissues (**Figure 2C**). The primer set P924/P923 was used to detect mRNA expressed from the *mGlp-1r* and *Glp-1r^−/−^* alleles (**Figure 2C**). By design, this set amplified the region between the murine 5′ UTR and mouse exon 3 and detected the presence of *Glp-1r* mRNA in both *mGlp-1r* and *Glp1r^−/−^* animals (**Figures 2D and 2G–H**). This primer set amplified a 275 bp band in *mGlp-1r* cDNA and a 372 bp fragment in cDNA generated from *Glp1r^−/−^* mice; the larger amplicon occurred as a result of mRNA processing, shown in Figure 2D. Housekeeping gene 36B4 was detected equally in all genotypes (**Figure 2C**). DNA sequencing of the 372 bp fragment from *Glp1r^−/−^* mice confirmed the mRNA transcript expressed in these animals does not code for a functional protein; this transcript does not contain intronic sequence and arises from the splice donor site upstream of the remaining LoxP site and the splice acceptor site located upstream of the intact mouse exon 2 (**Figure 2D**). The size difference between wild-type (*mGlp-1r*) and *Glp-1r^−/−^* sequence is 97 nucleotides, which corresponds to the length of exon 2; the splicing event that generates the fusion of human exon 2 and mouse exon 2 results in a frame-shift early in the *Glp-1r* open reading frame that gives rise to a predicted 98-aa truncated protein (**Figures 2E–F**).

**Figure 3:**
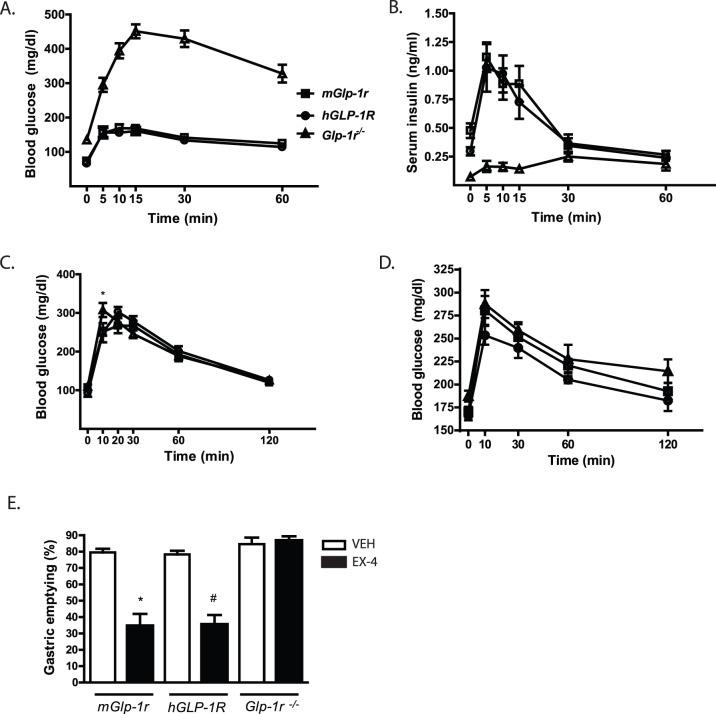
Glucose and mixed meal tolerance, and gastric emptying. (A) IPGTT. EX-4 (10 nmol/kg SC) was administered 30 minutes before glucose injection (2 g/kg glucose, IP injection). Both *mGlp-1r* and *hGLP-1R* mice experienced glucose lowering in response to EX-4, while the *Glp-1r^−/−^* mice were refractory to the glucose-lowering effect of EX-4. (B) Insulin secretion during the IPGTT was measured in *mGlp-1r*, *hGLP-1R,* and *Glp-1r^−/−^* mice. (C) OGTT with 2 g/kg glucose gavage in *mGlp-1r*, *hGLP-1R,* and *Glp-1r^−/−^* mice after an overnight fast. (D) Glucose excursion and during the MMTT in *mGlp-1r*, *hGLP-1R,* and *Glp-1r^−/−^* mice after a 4 hour fast (n = 7/group). (E) Gastric emptying in response to EX-4 (10 nmol/kg SC) in *mGlp-1r*, *hGLP-1R,* and *Glp-1r^−/−^* mice. All animals were fasted overnight and then treated with vehicle (VEH, white bars) or EX-4 (black bars) prior to food intake in order to measure the rate of gastric emptying after a meal. *p<0.001 VEH vs. EX-4 in *mGlp-1r* mice; ^#^p<0.001 VEH vs. EX-4 in *hGLP-1R* mice (n = 5–7 per group, one-way ANOVA with Bonferroni post-tests).

Body weights (*mGlp-1r* 24.3±0.7; *hGLP-1R* 22.2±0.4; and *Glp-1r^−/−^*23.8±0.8 g) and fasting blood glucose concentrations (*mGlp-1r* 91.8±9.3; *hGLP-1R* 106.3±9.9; and *Glp-1r^−/−^*110.3±8.6 mg/dl) were similar for each genotype. Because GLP-1R activation helps regulate glucose homeostasis by enhancing postprandial insulin secretion and delaying gastric emptying [33], [34], we performed studies to evaluate these processes. First, an IPGTT was done on all three genotypes to examine the glucose excursion profile 30 minutes after SC EX-4 administration (10 nmol/kg) (**Figure 3A**). As expected, the *mGlp-1r* and *hGLP-1R* mice responded with enhanced glucose lowering following EX-4 treatment compared to *Glp-1r ^−/−^* mice that showed no glucose lowering response to EX-4 during the IPGTT (**Figure 3A**). *Glp-1r^−/−^* mice had no insulinotropic response to EX-4 compared to the robust insulin secretion peaks seen in the *hGLP-1R* and *mGlp-1r* animals during the IPGTT (**Figure 3B**). Next, OGTTs conducted in *mGlp-1r*, *hGLP-1R,* and *Glp-1r ^−/−^* animals demonstrated no differences in glucose clearance among the groups (**Figure 3C**). However, since lipids and proteins are also potent stimulators of GLP-1 secretion [35], we also conducted a MMTT to assess the impact of GLP-1R ablation on postprandial glucose metabolism (**Figure 3D**). Blood glucose was measured in males given 10 ml/kg BW of Ensure Plus after a 4-hour fast, and glucose excursion during the MMTT did not differ among the three genotypes (**Figure 3D**).

Finally, we administered subcutaneous (SC) EX-4 to the three genotypes to assess GLP-1R-mediated gastric emptying. Both *mGlp-1r* and *hGLP-1R* mice treated with EX-4 displayed approximately 50% decrease in gastric emptying compared to vehicle-treated animals (PBS) (**Figure 3E**). However, the *Glp-1r ^−/−^* animals showed no decrease in gastric emptying in response to EX-4. Together, the data show that *hGlp1r* mice displayed comparable glucose homeostasis to wild-type *mGlp-1r* mice. Similar to previous reports evaluating *Glp-1r ^−/−^* mice, the model presented here does not respond to EX-4, consistent with the absence of the GLP-1R [5], [6], [36].

**Figure 4:**
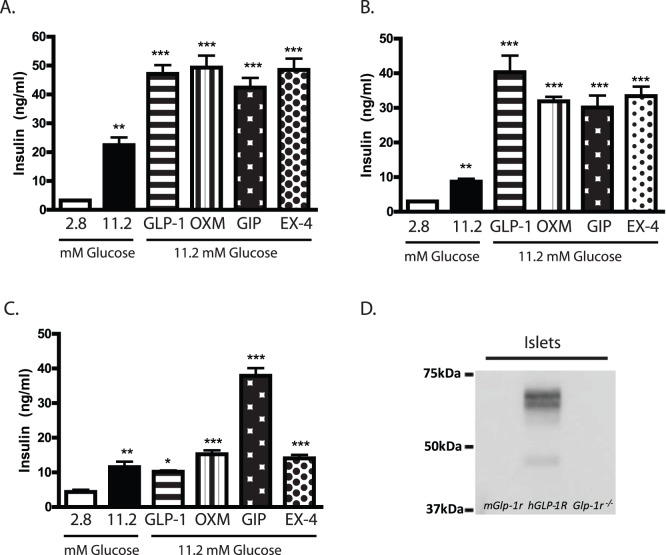
Insulin secretion and GLP-1R protein expression in pancreatic islets. (A) *mGlp-1r* and (B) *hGLP-1R* islets secreted insulin in response to high glucose (11.2 mM) compared to low glucose (2.8 mM) treatment, potentiated by GLP-1, OXM, GIP, and EX-4. (C) *Glp-1r^−/−^* islets also secreted insulin in response to high glucose, but no potentiation was observed with GLP-1, OXM, or EX-4 treatment. GIP-induced insulin secretion remained intact in *Glp-1r^−/−^* islets. (D) IP-WB of islets from *mGlp-1r*, *hGLP-1R*, and *Glp-1r^−/−^* mice showed FLAG expression in only the *hGLP-1R* islets. Hand-selected, size-matched islets were used for all insulin secretion assays, and comparisons are from high (11.2 mM) glucose: **p<0.01; ***p<0.001 (one-way ANOVA with Tukey’s multiple comparison test).

GLP-1R activation in β-cells of pancreatic islets enhances glucose-stimulated insulin secretion. To directly assess GLP-1R-dependent insulin secretion, islets were isolated from *mGlp-1r*, *hGLP-1R,* and *Glp-1r ^−/−^* mice, and static cultures of islets were treated with low or high glucose alone and in combination with GLP-1 (7–36)-NH_2_, oxyntomodulin (OXM), glucose-dependent insulinotropic peptide (GIP), or EX-4 (**Figures 4A–C**). Insulin concentrations in the medium were measured and compared across groups (**Figures 4A–C**). Both *mGlp-1r* and *hGLP-1R* islets showed increased insulin levels in the presence of high glucose (**Figures 4A–B**) and displayed significant increases in insulin secretion in response to high glucose in the presence of GLP-1, OXM, GIP, and EX-4 (**Figures 4A–B**). The *Glp-1r ^−/−^* islets also displayed glucose-stimulated insulin secretion, but only GIP enhanced insulin release in these cultures, consistent with the specific loss of GLP-1R signaling (**Figure 4C**). Further, the insulinotropic response of *Glp-1r^−/−^* islets to GIP was comparable to the effect of GIP in *mGlp-1r* and *hGLP-1R* islets, demonstrating that deletion of the GLP-1R does not impact the GIP-GIP receptor pathway (**Figure 4C**). IP-WB using an anti-FLAG antibody confirmed expression of hGLP-1R protein only in islets from *hGLP-1R* mice; GLP-1R protein migrated to various molecular weights due to glycosylation (**Figure 4D**).

### IHC

**Figure 5:**
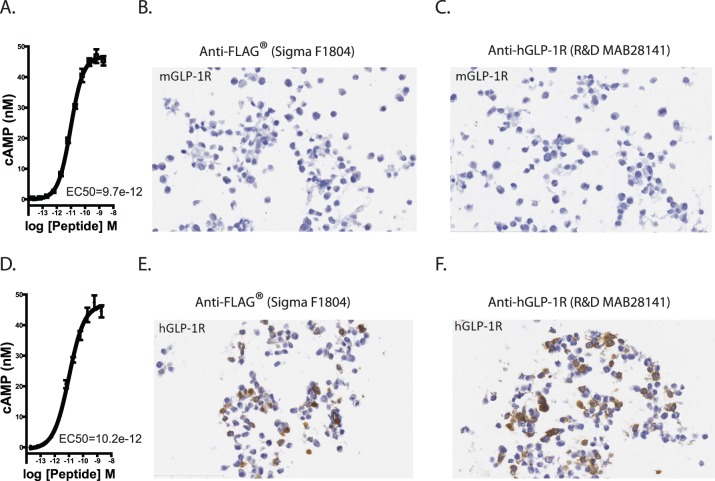
Validation of GLP-1R and FLAG antibodies. (A) *mGlp-1r* and (D) *hGLP-1R* were transiently expressed in HEK cells. cAMP accumulation in response to GLP-1 showed similar cAMP accumulation. HEK cells expressing mGLP-1R showed no (B) FLAG or (C) FLAG-tagged hGLP-1R expressing. HEK cells show stained for (E) FLAG and (F) hGLP-1R protein after transient transfection.

HEK cells were transfected with FLAG-tagged *hGLP-1R* or untagged, murine *Glp-1r* constructs to evaluate commercially available GLP-1R and FLAG antibodies. cAMP assays confirmed both expression and activity of mGLP-1R and FLAG-tagged hGLP-1R proteins (**Figures 5A and 5D**). IHC for FLAG showed that *mGlp-1r*-expressing cells did not stain for FLAG (**Figure 5B** *)*. Furthermore, when these cells were stained with antisera specific for human GLP-1R (R&D MAB 28141), we did not detect any signal, suggesting this antibody does not recognize mGLP-1R (**Figure 5C**). Conversely, *hGLP-1R*-transfected cells stained strongly for the FLAG epitope (**Figure 5E**), and hGLP-1R was detected by the hGLP-1R-specific antibody from R&D (**Figure 5F**). We evaluated additional GLP-1R antisera (Abcam 39072) which showed only non-specific staining in both *mGlp-1r* and *hGLP-1R* tissues (data not shown), consistent with previous work demonstrating non-specific staining of this antibody [16].

**Figure 6:**
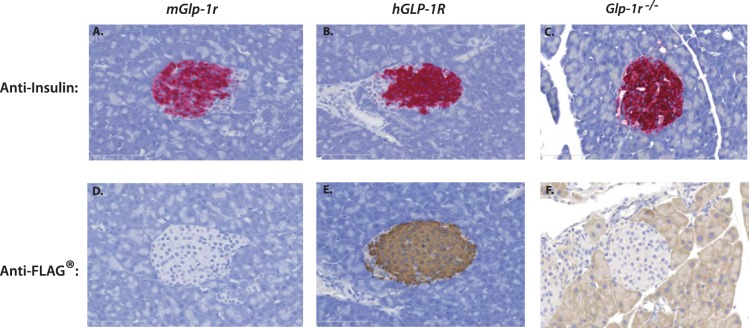
IHC of pancreata from *mGlp-1r*, *hGLP-1R,* and *Glp-1r^−/−^* mice. Pancreata were stained for insulin and showed positive staining in the β-cells of (A) *mGlp-1r*, (B) *hGLP-1R,* and (C) *Glp-1r^−/−^* islets. FLAG staining was also performed and (E) *hGLP-1R* islets stained positive for FLAG with none detected in (D) *mGlp-1r* and (F) *Glp-1r^−/−^* islets.

**Figure 7:**
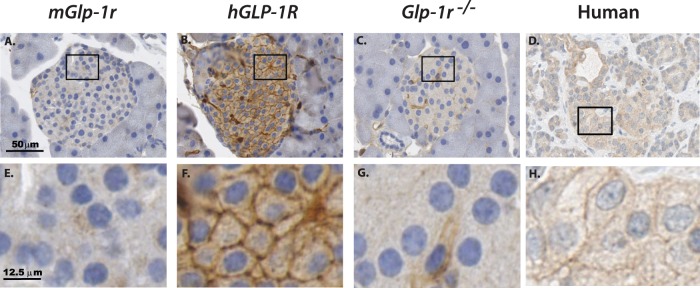
IHC for human GLP-1R expression. Islets from (A) *mGlp-1r*, (B) *hGLP-1R,* (C) *Glp-1r^−/−^* mice and (D) human pancreas were stained using an antibody specific for human GLP-1R, and only the (B) *hGLP-1R* islets and (D) human pancreas showed positive signal. Increased magnification showed that while (E) *mGlp-1r* and (G) *Glp-1r^−/−^* islets display no hGLP-1R expression, the (F) *hGLP-1R* islets and (H) human islets show plasma membrane staining for the human GLP-1R.

After validation of the anti-FLAG and anti-hGLP-1R antibodies, IHC was performed on pancreata harvested from *mGlp-1r*, *hGLP-1R,* and *Glp-1r^−/−^* mice. Sections from all animals showed strong insulin staining within the core of islets (**Figures 6A–C**). Staining for FLAG in pancreatic sections of the three genotypes showed strong signal within β-cells of the *hGLP-1R* mice and none in *mGlp-1r* or *Glp-1r^−/−^* animals (**Figures 6D–F**). Human GLP-1R was present only in the *hGLP-1R* islets, and therefore, the anti-hGLP-1R antibody did not show staining in *mGlp1-r* or *Glp-1r^−/−^* islets (**Figures 7A–C**). Upon higher magnification, hGLP-1R was observed at the plasma membrane of β-cells in *hGLP-1R* islets, confirming proper sub-cellular localization (**Figures 7E–G**). We also performed IHC on human pancreas with the hGLP-1R-specific antibody and showed GLP-1R expression within the islets and its localization at the plasma membrane (**Figures 7D and H**).

**Figure 8:**
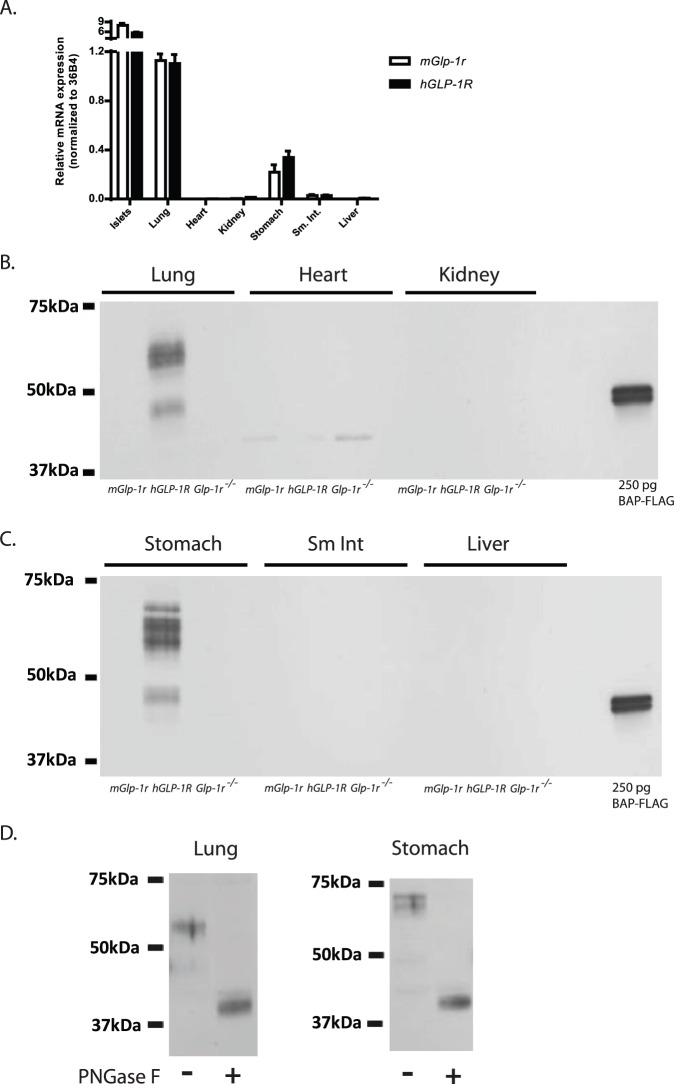
GLP-1R mRNA and protein expression in vivo. (A) qPCR analyses showed abundant expression in islets, lung, and stomach in both *mGlp-1r* and *hGLP-1R* mice. (B)–(C) Western blots using anti-FLAG affinity purification and immunoblotting showed abundant expression of the FLAG-tagged human GLP-1R in the lung and stomach of the *hGLP-1R* mice. Moreover, there was no FLAG-tagged GLP-1R detected in whole heart, kidney, small intestine (sm. int.), or liver of *hGLP-1R* mice. Using the FLAG signal as a surrogate for GLP-1R protein, no expression was observed for the receptor in *Glp-1r^−/−^* tissues, indicating that LoxP-mediated deletion of the *hGLP-1R* gene was successful. Purified bacterial alkaline phosphatase (BAP)-FLAG was included in gels as a control for the FLAG antibody. (D) Treatment of samples with PNGase F caused the multiple bands to collapse together from GLP-1R deglycosylation, resulting in a band of smaller molecular weight than the glycosylated form of the receptor.

In order to ascertain the relative levels of GLP-1R mRNA and protein expression *in vivo*, the following tissues were harvested from *mGlp-1r*, *hGLP-1R*, and *Glp-1r^−/−^* mice: heart, islets, lung, kidney, stomach, small intestine, and liver (**Figure 8A**). qPCR showed abundant GLP-1R mRNA in islets, lung, and stomach, with similar expression levels observed in both *hGLP-1R* and *mGlp-1r* tissues (**Figure 8A**). qPCR analyses of both hypothalamus and brain stem of *mGlp-1r* and *hGLP-1R* mice revealed moderate GLP-1R mRNA expression, with lower expression detected in cerebral cortex and pituitary (data not shown). We also detected low GLP-1R mRNA expression in heart tissue (**Figure 8A**).

We first performed traditional WBs on tissue lysates from *mGlp-1r*, *hGLP-1R*, and *Glp-1r^−/−^* with an anti-FLAG antibody, but a specific signal at the predicted molecular weight for GLP-1R was not detected (data not shown). Therefore, an IP-WB method was implemented using anti-FLAG antibody-conjugated magnetic beads to first pull down the FLAG-tagged protein, followed by cross-blot using a different anti-FLAG antibody (**Figures 4D** **,** **8B–D**). Similar to the *hGLP-1R* islets (**Figure 4D**), lung and stomach tissue (**Figure 8D**) showed bands of varying molecular weight that likely represent glycosylated forms of GLP-1R [37]–[39].

Using two monoclonal anti-FLAG antibodies to detect the presence of the GLP-1R, we showed that the most abundant expression of GLP-1R occurs in islets, lung, and stomach (**Figures 4D** **and** **8B–C**). The *Glp-1r^−/−^* tissues did not show FLAG expression, confirming the absence of functional GLP-1R protein. FLAG-tagged GLP-1R was not detected in whole heart, kidney, small intestine, or liver of *hGLP-1R* mice, indicating that GLP-1R protein expression is absent or below the limit of detection of our anti-FLAG method (**Figures 8B–C**). The three genotypes show a non-specific band in heart (**Figure 8B**). In addition to islets, the glycosylated forms of GLP-1R were also present in both lung and stomach of the *hGLP-1R* mice; incubation of PNGase F with these samples collapsed the banding pattern, giving rise to a single band of lower molecular weight (**Figure 8D**).

## Discussion

In this study, we show that replacement of the murine GLP-1R with human GLP-1R results in a similar metabolic phenotype. *Ex vivo* assays of islets isolated from both genotypes demonstrate comparable insulinotropic responses to glucose, as well as enhancement of GLP-1-, GIP-, OXM-, and EX-4-stimulated insulin secretion. Further, the targeting construct used for the knock-in model enabled creation of *Glp-1r^−/−^* mice derived via Cre-mediated deletion of the *hGLP-1R*. Generation of whole-body *Glp-1r^−/−^* mice by *Rosa26Cre*-mediated excision confirms utility of the LoxP sites incorporated within the knock-in locus, a feature that will allow conditional gene deletion strategies in the future. Characterization of this *Glp-1r^−/−^* model demonstrates loss of GLP-1R-dependent insulin secretion and gastric emptying; these animals display no response in glucose lowering after EX-4 treatment during IPGTTs, consistent with the established role of the GLP-1R [1], [33].

The emerging controversy surrounding the safety implications of GLP-1 analogues and DPP4 inhibitors [40]–[42] underscores the importance of a thorough characterization of GLP-1 biology. Establishing another *Glp-1r* ^−/−^ model using a different gene deletion strategy is important in fully characterizing GLP-1R function, especially because the targeting approach used for the first model allowed expression of a 186 aa form of GLP-1R [16], [43]. Data from experiments evaluating our new *Glp-1r* ^−/−^ model vary slightly with the published results characterizing the initial *Glp-1r* null mouse [43]. The first global *Glp-1r* ^−/−^ mouse, reported by Scrocchi *et al*, showed that whole-body *Glp-1r^−/−^* mice generated on the CD1 background had higher basal fasting glucose levels and impaired oral glucose tolerance compared to wild-type littermates [43]. A subsequent study describing the double incretin knock-out (DIRKO) mice showed that male *Glp-1r^−/−^* mice are only mildly glucose intolerant while females exhibited normal glucose tolerance after being backcrossed for five generations onto the C57BL/6 strain [5]. In 2013, Wilson-Peréz et al reported another *Glp-1r^−/−^* mouse that was also generated by Cre-LoxP-mediated gene deletion on the C57BL/6 background, and showed that on a high-fat diet, loss of the *Glp-1r* impairs glucose clearance and insulin secretion in response to a mixed meal challenge; however, the authors did not report findings on intraperitoneal or oral glucose tolerance tests [29]. Our studies using the newly generated male *Glp-1r^−/−^* mice show similar oral glucose and mixed meal tolerance compared to *mGlp-1r* mice fed a chow diet, consistent with the phenotype observed in female *Glp-1r^−/−^* mice reported by Hansotia et al [5]. Further, the lack of EX-4-induced insulin secretion in the *Glp-1r^−/−^* mice during the IPGTT, as well as the absence of GLP-1R protein and hGLP-1R mRNA, confirm complete *Glp-1r* ablation in the *Glp-1r^−/−^* mouse model reported here. Subtle differences in glucose metabolism between *Glp-1r^−/−^* mice in our study and those reported previously [5], [29] may be attributable to other factors such as differences in diets or housing conditions.

Recent work aimed at studying GLP-1R function in various tissues revealed concerns about data generated using the existing commercial antisera raised against rodent and human GLP-1R due to the inability of antibodies to specifically detect GLP-1R protein [16], [24]. Panjwani *et al* tested three antibodies using wild-type and *Glp-1r^−/−^* lung tissue extracts, and each failed to detect GLP-1R [16]. In this regard, inclusion of a FLAG epitope at the C-terminus of the *hGLP-1R* gene in the knock-in construct allows use of well-characterized anti-FLAG antibodies to examine tissue expression of GLP-1R protein. Further, the work here shows an R&D Systems antibody specifically detects human GLP-1R protein via IHC in HEK cells expressing hGLP-1R as well as in islets of the *hGLP-1R* knock-in mice and human pancreas. Consistent with these results, FLAG staining shows positive signal in islets from *hGLP-1R* but not in tissues from *mGlp-1r* or *Glp-1r^−/−^* mice.

The use of whole-body *Glp-1r^−/−^* mice and GLP-1R agonists such as EX-4 have helped establish many of the physiological roles of the GLP-1R, but determining the whole-body expression pattern of GLP-1R protein is needed to understand specific actions of GLP-1 on various organ systems. Glp-1r mRNA expression has been reported in the pancreas, brain, stomach, lung, small intestine, and colon of rats and mice [21]–[23]. RT-PCR/Southern blot analyses indicates lung has particularly high Glp-1r mRNA levels, as well as the body of the stomach, the small intestine, and the pancreas [23], while whole heart, kidney, and liver have extremely low or nearly undetectable levels of Glp-1r mRNA [22], [23]. The mRNA data presented here are consistent with previous reports on islets, lung, and stomach as being highly enriched for GLP-1R, and our results are further substantiated by western analyses showing strong GLP-1R protein signal in islets, lung, and stomach. We also found low Glp-1r mRNA expression in heart, which supports recent findings on the role of GLP-1R in atrial function [11]. Due to the lack of anti-GLP-1R antisera for immunoblotting, the western results are only possible because of the anti-FLAG technique employed here. Strategies incorporating use of FLAG-tagged proteins for *in vivo* tissue expression analyses have been used previously [44]–[46]. However, most of these efforts used transgenic systems that promote high expression of tagged proteins. Our humanized mouse model allows investigations of the tissue-specific expression patterns of the GLP-1R under the normal control of the murine *Glp-1r* locus. In this light, it is important to note that an affinity purification step was required to enrich for the GLP-1R protein before the western blot. Detecting GLP-1R in islets, lung, and stomach in *hGLP-1R* but not in *Glp-1r^−/−^* tissues emphasizes the need to routinely employ rigorous controls when attempting to draw conclusions based on western blot data from poorly characterized antibodies. For example, while some reports showing GLP-1R protein expression in pancreatic β-cells from both mouse and rat have used *Glp-1r^−/−^* mouse tissues as negative controls [47], other reports (using different antibodies) claiming GLP-1R in lung, cardiac, and vascular tissues have not been similarly controlled [48]. GLP-1R protein has also been detected using WB in pig kidney tubules, but again, in a study that lacked thorough validation of the GLP-1R antibody [49]. Our humanized mouse model will allow a more comprehensive investigation of expression patterns of the GLP-1R, and thus, may shed more light into its functions in extra-pancreatic tissues.

Although much is now known about the role of GLP-1 in controlling glucose metabolism, improving our understanding of the molecular mechanisms that regulate GLP-1R function in β-cells and other tissues may enable development of improved GLP-1R-based therapies. An emerging area in GPCR biology is identifying partner or accessory proteins and understanding how GPCR interacting proteins help control signaling. Studies using *in vitro* systems incorporating GPCRs tagged with FLAG or HA have identified interacting protein partners [50]–[52]; tagging the GLP-1R *in vivo* with FLAG may allow similar approaches. Thus, the FLAG epitope should enable biochemical study of the GLP-1R through IP and mass spectrometry analyses. In summary, we present the first humanized *GLP-1R* knock-in mouse, a model that allows the exploration of incretin biology of the hGLP-1R in mice. The *Glp-1r^−/−^* mouse generated here offers a complementary tool with which to further characterize GLP-1 biology. Together, the *hGLP-1R* and *Glp-1r^−/−^* mice will enable targeted biochemical study and broad tissue expression analysis of GLP-1R protein.

## Supporting Information

Figure S1 Genotyping strategy and primer sets for wild-type (*mGlp-1r*) and humanized knock-in (*hGLP-1R*) lines. The *mGlp-1r* PCR product is a single band of 138 bp; the heterozygous PCR product shows the wild-type band (138 bp) and the *hGLP-1R* band of 482 bp. The *hGLP-1R* PCR product is a single 482 bp band.
(EPS)

Figure S2 Genotyping strategy and primer sets for the *Glp-1r^−/−^* line. The *Glp-1r^−/−^* line shows a single 221 bp band. The wild-type band of 138 bp with the 221 bp *Glp-1r^−/−^* band denotes *Glp-1^+/−^* mice.
(EPS)

Table S1 Primer and probe sequences used in this study (5′–3′).
(EPS)
