# Correction: A Novel Humanized GLP-1 Receptor Model Enables Both Affinity Purification and Cre-LoxP Deletion of the Receptor

**DOI:** 10.1371/journal.pone.0132875

**Published:** 2015-07-14

**Authors:** Lucy S. Jun, Aaron D. Showalter, Nosher Ali, Feihan Dai, Wenzhen Ma, Tamer Coskun, James V. Ficorilli, Michael B. Wheeler, M. Dodson Michael, Kyle W. Sloop

The expected band size in Fig 2D and the cartoon graphic in Fig 2F are incorrect. The authors have provided a corrected version of Fig 2 here.

**Fig 2:**
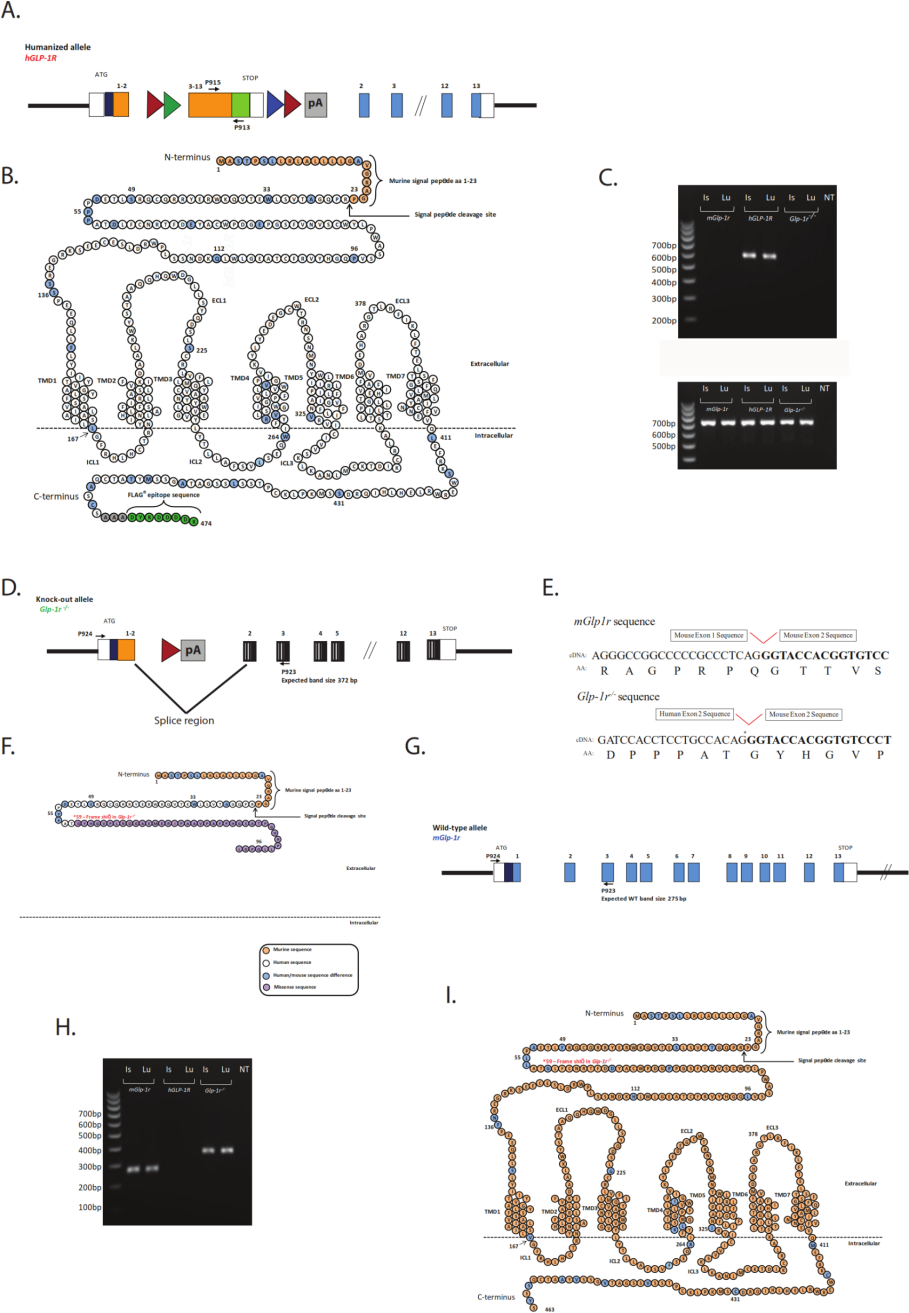
Reverse transcription for PCR validation of *hGLP-1R*, *mGlp-1r* and *Glp1r* ^*−/−*^ lines. (A) A schematic of the gene that is expressed in the *hGLP-1R* mice. (B) The protein that is produced from this gene is a fusion of the mouse signal peptide (beige residues) and the human GLP-1R protein (white residues). The blue residues are those that differ between mouse and human GLP-1R. The signaling peptide is cleaved, leaving behind a human GLP-1R protein containing the C-terminal FLAG epitope (green residues). (C) cDNA was generated from total RNA isolated from islet and lung of *hGLP-1R*, *mGlp-1r*, and *Glp1r* ^***−/−***^ mice for RT-PCR. The 5′ P915 primer annealed in human exon 8, while the 3′ P913 primer annealed to the FLAG region, a unique site within the *hGLP-1R* gene. This PCR product is a 588 bp fragment only observed in the *hGLP-1R* mice. (D) A schematic of the gene that is expressed in the *Glp-1r* ^***−/−***^ mice. (E) Once the splice event occurs between human exon 2 and mouse exon 2, a frame-shift mutation nullifies downstream protein expression. (F) The final protein product in *Glp-1r* ^***−/−***^ mice is a 98-aa truncation mutant. The first 36 aa’s of the mature protein encode a fraction of the GLP-1R extracellular domain, and the remaining 40 aa’s constitute missense sequence that shows no similarity to known proteins. The RT-PCR and DNA sequence analyses of the *Glp-1r* ^***−/−***^ gene product demonstrate the *Glp-1r* ^***−/−***^ mouse does not code for a functional GLP-1R. (G) A schematic of the wild-type (*mGlp-1r*) gene. (H) Using the same primer pair, PCR products from *Glp-1r* ^***−/−***^ (372 bp) and *mGlp-1r* (275 bp) mice differ in size by 97 bp. (I) The wild-type GLP-1R protein is 463 aa’s including the signaling peptide.
